# Systemic metastatic retinal lymphoma masquerading as granulomatous uveitis

**DOI:** 10.3205/oc000266

**Published:** 2026-02-06

**Authors:** Ayşe Yağmur Kanra, Saniye Üke Uzun

**Affiliations:** 1 Şişli Hamidiye Etfal Training and Research Hospital, Istanbul, Turkey; 2 Etlik Public Hospital, Ankara, Turkey

**Keywords:** adrenal lymphoma, optic nerve infiltration, masquerading syndromes, vitreoretinal involvement

## Abstract

**Objective:**

To highlight the importance of considering systemic metastatic retinal lymphoma (SMRL) in the differential diagnosis of atypical uveitis and to emphasize the need for a comprehensive diagnostic and treatment approach for optimal patient outcomes.

**Methods:**

Medical records and imaging of the patient were retrospectively reviewed.

**Results:**

A 61-year-old man presented with blurred vision, initially diagnosed as granulomatous uveitis following cataract surgery. Further ocular examination revealed optic nerve infiltration and retinal white infiltrates, prompting additional systemic evaluation. Imaging and biopsy confirmed a diagnosis of primary adrenal non-Hodgkin lymphoma with ocular involvement. The patient underwent R-CHOP chemotherapy and autologous stem cell transplantation during the COVID-19 pandemic, achieving systemic remission. However, due to pandemic-related logistical challenges, he declined further ocular treatment. At 1.5-year follow-up, his final visual acuity was 20/25 in the right eye and 20/125 in the left eye, with persistent cystoid macular edema in the left eye.

**Conclusion:**

This case underscores the importance of recognizing SMRL as a potential cause of atypical uveitis, particularly in the presence of optic nerve and retinal infiltrates. It highlights the need for early diagnosis and a multidisciplinary approach, integrating systemic and ocular management to improve patient outcomes.

## Introduction

The term “masquerade syndrome” refers to disorders that mimic chronic uveitis. It was first introduced in ophthalmic literature in 1967 to describe conjunctival carcinoma presenting as chronic conjunctivitis [1]. While some non-malignant causes, such as intraocular foreign bodies, retinal degenerations, and pigment dispersion syndrome, can lead to masquerade syndromes, the majority are associated with malignant processes, necessitating early diagnosis and timely intervention.

Intraocular lymphoproliferative diseases can manifest as primary intraocular lymphoma (PIOL) or as secondary metastases from systemic lymphoma. The uvea is the most common ocular site of metastatic involvement, while systemic metastatic retinal lymphoma (SMRL) is a rare entity, accounting for only 5% of reported cases [2], [3]. In such cases, optic nerve lymphomatous infiltration is an even more uncommon presentation [4].

Here, we present a case of primary adrenal non-Hodgkin lymphoma with ocular involvement, initially misdiagnosed as granulomatous uveitis, which was later confirmed as SMRL with optic nerve infiltration. This case highlights the importance of considering SMRL in atypical uveitis presentations and the challenges of systemic and ocular management, particularly in complex clinical settings.

## Case description

A 61-year-old man presented with a 3-month history of blurred vision that started in the first month after cataract surgery. At the initial visit, the patient had a visual acuity of 20/25 in the right eye and 20/200 in the left eye. Slit-lamp biomicroscopy revealed bilateral granulomatous anterior uveitis, with microgranulomatous keratic precipitates predominantly located on the inferior corneal endothelium. The anterior chamber reaction was graded as +1 in both eyes. Posterior segment evaluation showed mild vitreous inflammation in the right eye, with trace to +1 vitreous cells and minimal haze. The left eye exhibited severe vitreous inflammation, characterized by +4 vitreous cells and dense vitreous haze, which significantly impaired visualization of the fundus. Due to the obscured fundus view, B-scan ultrasonography was performed and revealed moderate opacities without signs of retinal detachment and mass. 

**Figure 1:**
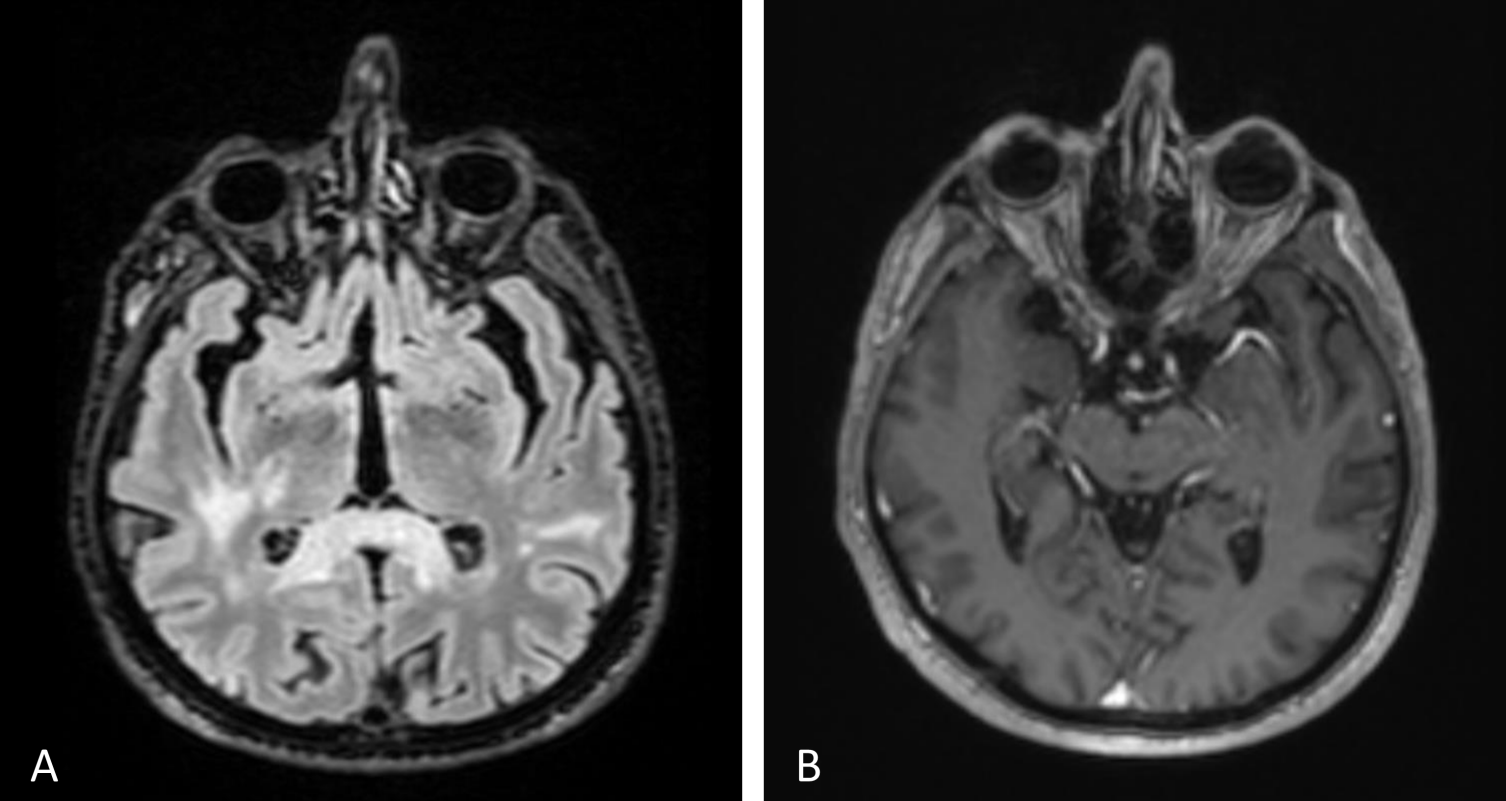
Contrast and non-contrast cranial MRI sections from systemic investigation. The left optic nerve appeared bulkier and larger, with multiple nodular lesions visible in the brain parenchyma (A–B). The choroidal and retinal layers were diffusely thickened and showed greater enhancement in the left eye compared to the right (B).

The systemic workup was suggestive of a granulomatous process, including lymphopenia, high CRP level, 2-fold increase in angiotensin-converting enzyme (ACE), purified protein derivative (PPD) anergic, Quantiferon+, and findings consistent with previous tuberculosis (TB) on thorax computed tomography (CT). However, multiple parenchymal nodular lesions were observed in cranial and orbital MR scans, along with thickening and enhancement of the left optic disc (Figure 1 (Fig. 1)). These findings are indicative of an infiltrative process, which corresponds to the patient’s systemic condition. The patient has experienced a 7–8 kg unintentional weight loss over the past few months, along with fatigue and a history of smoking 30 packs per year. We did not find any evidence of malignancy in the cerebrospinal fluid sample or vitreous aspirate. 

Abdominal CT revealed a contrast-enhanced mass in the right adrenal gland. Oncological positron emission tomography (PET) showed a 69×49 mm well-circumscribed adrenal mass with intense fluorodeoxyglucose (FDG) uptake and central hypometabolism. Hyperdense lymphadenopathies (LAPs) (up to 14 mm) with increased FDG uptake, suggestive of a granulomatous process, were observed in the mediastinum, lower paratracheal, aorticopulmonary window, subcarinal, and hilar regions. CT-guided biopsy confirmed CD20+ large B-cell lymphoma. 

**Figure 2:**
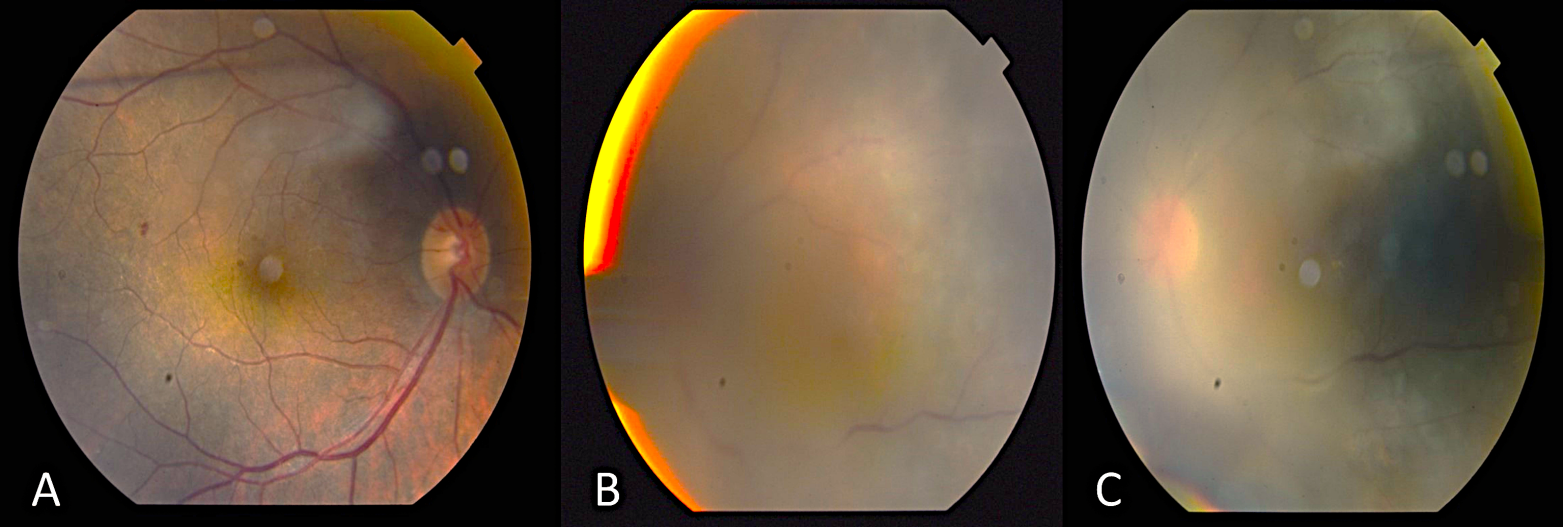
Color photograph of both fundi post systemic steroid treatment with isoniazid prophylaxis The optic nerve appeared hyperemic and infiltrated, and the retina demonstrated diffuse white infiltrates with associated generalized retinal thickening, vascular sheathing, and venous beading, predominantly in the left eye (A–C).

**Figure 3:**
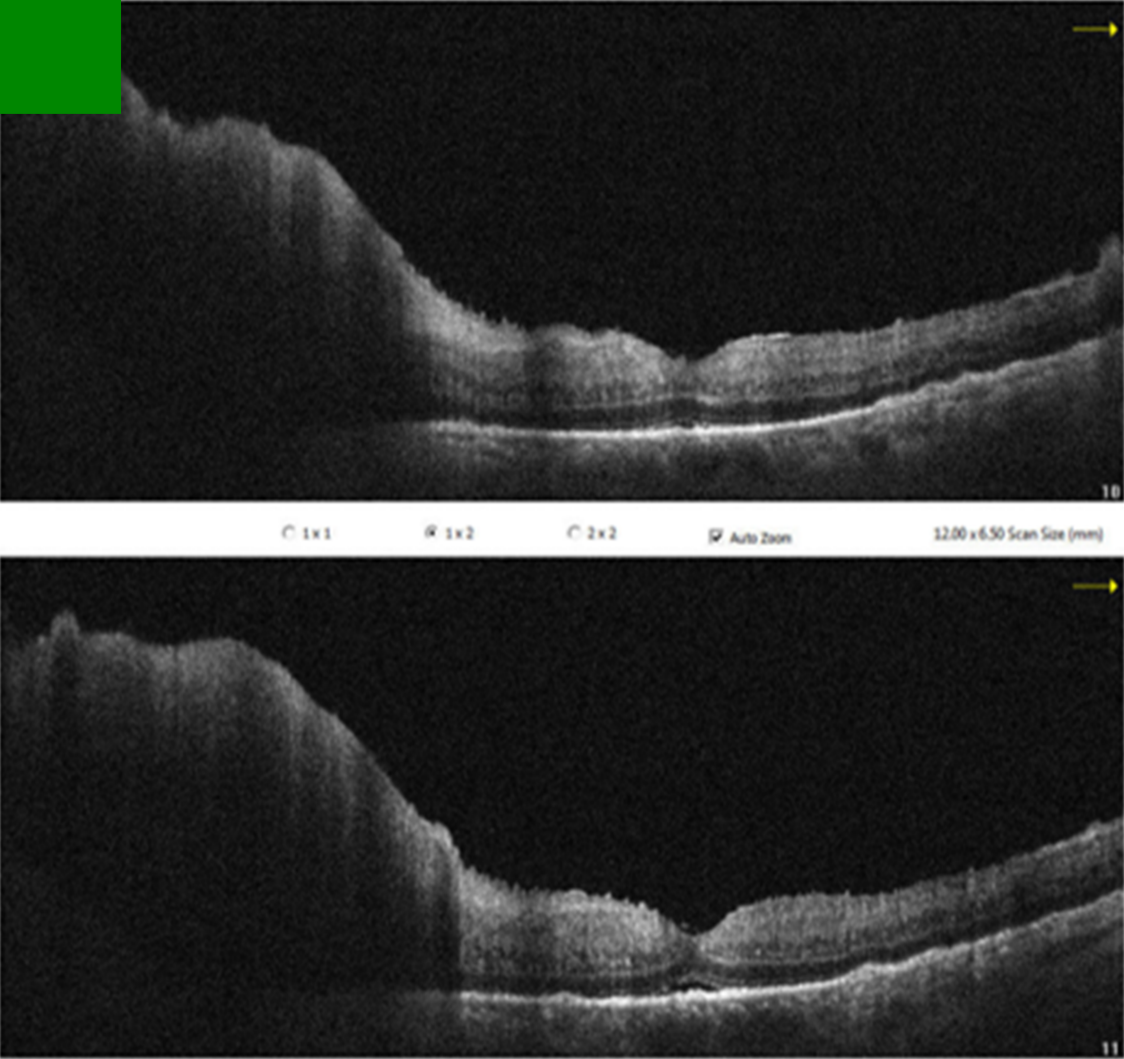
Pre-treatment OCT sections of the left eye OCT revealed irregularities and multiple subretinal hyperreflective deposits at the level of the retinal pigment epithelium and Bruch’s membrane. These irregularities and deposits were predominantly observed in the left eye, accompanied by severe optic disc edema.

**Figure 4:**
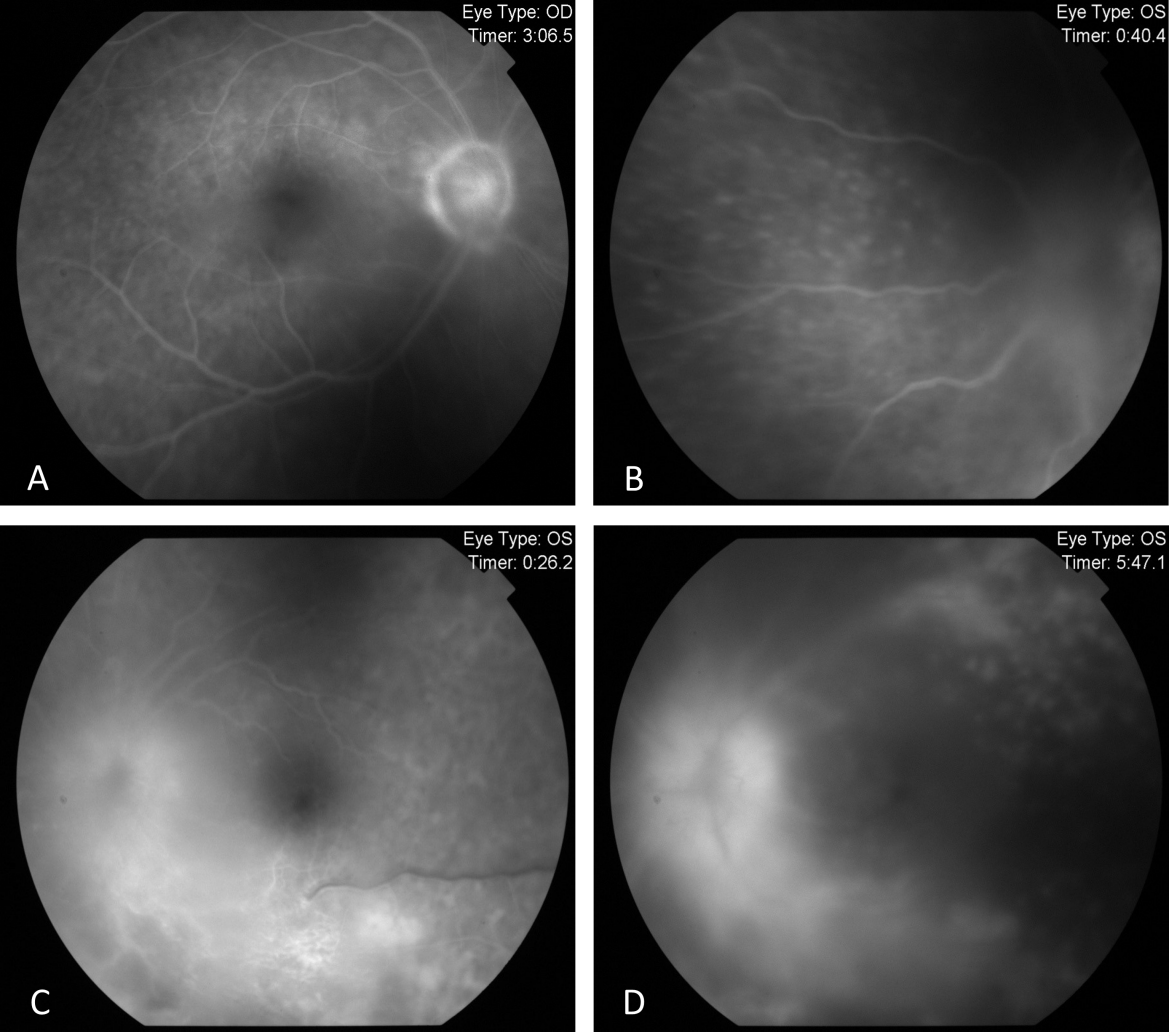
Pre-treatment FFA images FFA exhibited indistinct granular hyperfluorescence and mild staining in the optic nerve of the right eye (A). In the left eye, FFA revealed early and dense leakage in the optic nerve, accompanied by venous segmentations, generalized granular hyperfluorescence, vasculitis, and areas of capillary dropout (B–D).

Since the visual acuity decreased to hand motion during systemic investigations, systemic steroid treatment was initiated with isoniazid (INH) prophylaxis. Following treatment, the vitreous inflammation in the left eye improved from +4 to +2 cells, allowing improved visualization of the posterior segment. The optic nerve appeared hyperemic and infiltrated, and the retina demonstrated diffuse white infiltrates with associated generalized retinal thickening, vascular sheathing, and venous beading, predominantly in the left eye (Figure 2 (Fig. 2)). Optical coherence tomography (OCT) showed irregularities and multiple subretinal hyperreflective deposits in the retinal pigment epithelium and Bruch membrane level, which were predominantly present in the left eye and severe optic disc edema (Figure 3 (Fig. 3)). Fundus fluorescein angiography (FFA) revealed indistinct granular changes and mild disc staining in the right eye. Active lesions were characterized by a typical early hypofluorescent pattern observed in the retinal infiltrates, with late hyperfluorescence containing dilated retinal vessels, diffuse pinpoint leakage at the infiltrative border in the left eye and areas of capillary dropout were observed (Figure 4 (Fig. 4)). 

**Figure 5:**
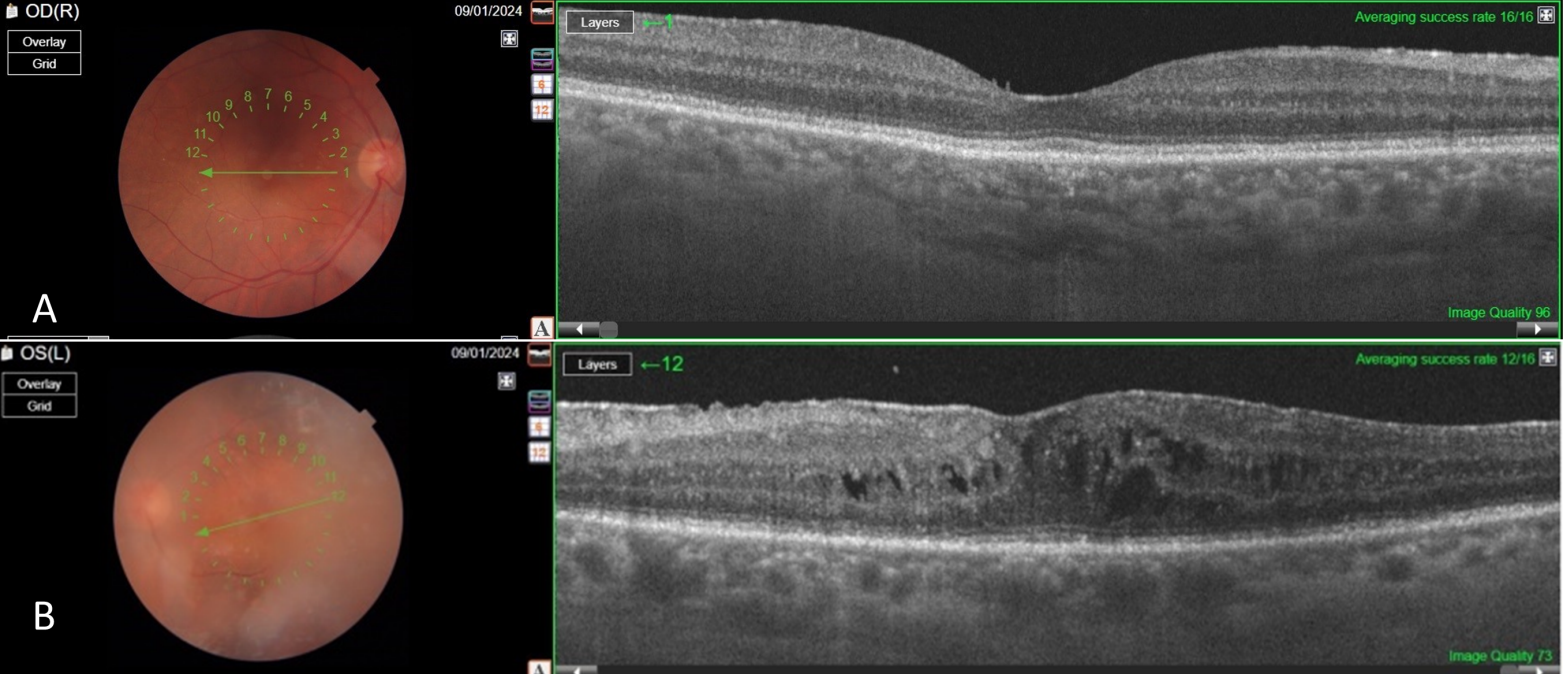
Post-treatment OCT images The findings regressed in both eyes compared to the pre-treatment period (A–B). However, vitreous opacity and cystoid macular edema were observed, only in the left eye (B). Additionally, an epiretinal membrane induced by bilateral vitreous inflammation was noted (A–B)

The patient completed R-CHOP protocol chemotherapy during the COVID-19 pandemic and underwent autologous stem cell transplantation, achieving systemic disease remission. However, the patient declined further treatment for brain and ocular involvement due to repeated COVID positivity and the challenges of treatment. At the 1.5-year follow-up, the right eye retained a visual acuity of 20/25, while the left eye was 20/125. Biomicroscopy showed quiet anterior segments in both eyes. However, the left eye exhibited residual +1 vitreous cells, cystoid macular edema, and an epiretinal membrane consistent with chronic inflammation (Figure 5 (Fig. 5)).

## Discussion

Primary adrenal non-Hodgkin’s lymphoma may arise from hematopoietic tissue in the adrenal gland. In this case, lymphopenia, elevated CRP, increased ACE, PPD anergy, Quantiferon positivity, and thorax CT findings consistent with prior TB suggested a granulomatous process. However, imaging revealed multiple nodular lesions in the brain and adrenal gland, raising suspicion of metastatic disease. Pathology from adrenal biopsy confirmed primary adrenal non-Hodgkin’s lymphoma. Although the vitreous biopsy and cerebrospinal fluid (CSF) sampling did not yield malignant cells, this is a known limitation, as up to 60–70% of such biopsies may be non-diagnostic [5]. IL-10/IL-6 cytokine ratios, although helpful in differentiating PIOL from chronic uveitis, were not available in our setting. Notably, this marker is less reliable in SMRL, particularly in T-cell-derived lymphomas, where IL-10 levels may remain low. B-cell-derived SMRLs, like in our case, may exhibit higher IL-10 levels but remain inconsistent for sole reliance [6].

Metastasis of systemic lymphoma to the optic nerve is rare, with only one reported case out of nearly 6,000 cases [7]. Vitreoretinal involvement from non-central nervous system metastasis is also reported in only 5% of cases [2], [3]. While vitreous aspirates can detect lymphomatous cells in up to 40% of ocular lymphoma cases, vitreous biopsy remains the gold standard for diagnosis [8]. Typically, retinal involvement in lymphoma manifests as numerous subretinal orange-yellowish infiltrates which have the capacity to expand and conjoin over time, ultimately generating a sizable subretinal mass. Histopathologic evaluations have demonstrated these infiltrates to be deposits of lymphoproliferative cells located between the retinal pigment epithelium (RPE) and Bruch membrane [8]. Accordingly, there were diffuse white infiltrates in the retina and OCT showed irregularities and multiple subretinal hyperreflective deposits in the retinal pigment epithelium and Bruch membrane level, which were mostly seen in the left eye. We did not find any evidence of malignancy in the cerebrospinal fluid sample or vitreous aspirate. 

Bedaiwi et al. [9] documented an instance of metastatic T-cell adrenal lymphoma in which the patient experienced intraocular manifestations. Specifically, the individual exhibited symptoms of anterior uveitis characterized by pinkish hypopyon and posterior synechia. Notably, a fundus examination of the patient’s left eye revealed the presence of whitish retinal infiltrate across multiple areas, however, neither vitritis nor optic disc edema were observed. Galletero Pandelo et al. [10] presented a report detailing the clinical features and progression of a patient diagnosed with primary adrenal lymphoma that had infiltrated the vitreous and optic nerve. The patient initially exhibited bilateral vitritis and unilateral optic disc edema, both of which demonstrated a favorable response to systemic chemotherapy and intravitreal administration of methotrexate (MTX). However, following a brief follow-up period, these lesions recurred, and brain metastases emerged, ultimately resulting in the patient’s demise. This case is very similar to the ophthalmic findings of our case. However, brain metastasis was present at the time of diagnosis in our case.

In previously reported case series, patients with systemic vitreoretinal lymphoma (VRL) have been treated with a combination of systemic chemotherapy and localized ocular therapies. For instance, a study by Pulido et al. reviewed 55 patients with vitreous specimens, identifying six cases where systemic lymphoma involved the vitreous body. These patients underwent systemic chemotherapy, and in some instances, adjunctive localized treatments such as intravitreal chemotherapy or ocular radiotherapy were administered, leading to varying degrees of ocular disease control [11]. Similarly, a comprehensive review by Chan et al. discussed the management of intraocular lymphomas, including SMRL. The authors highlighted that treatment often involves systemic chemotherapy combined with localized ocular treatments, such as intravitreal methotrexate injections or ocular radiotherapy, to effectively address both systemic and ocular manifestations of the disease [12]. 

## Conclusion

In our case, the patient declined ocular and central nervous system-specific treatments due to repeated COVID-19 positivity and pandemic-related challenges, leading to disease management with systemic therapy alone. This highlights the importance of considering SMRL in atypical uveitis presentations and emphasizes the need for comprehensive treatment strategies, including both systemic and localized therapies, to optimize outcomes in SMRL.

## Notes

### Patient consent

The patient described in the case report has given his informed consent for publication.

### Competing interests

The authors declare that they have no competing interests.
